# Pentamidine Dosage: A Base/Salt Confusion

**DOI:** 10.1371/journal.pntd.0000225

**Published:** 2008-05-28

**Authors:** Thomas P. C. Dorlo, Piet A. Kager

**Affiliations:** Center for Infection and Immunity Amsterdam (CINIMA), Division of Infectious Diseases, Tropical Medicine and AIDS, Academic Medical Center, University of Amsterdam, Amsterdam, The Netherlands; Swiss Tropical Institute, Switzerland

## Abstract

Pentamidine has a long history in the treatment of human African trypanosomiasis (HAT) and leishmaniasis. Early guidelines on the dosage of pentamidine were based on the base-moiety of the two different formulations available. Confusion on the dosage of pentamidine arose from a different labelling of the two available products, either based on the salt or base moiety available in the preparation. We provide an overview of the various guidelines concerning HAT and leishmaniasis over the past decades and show the confusion in the calculation of the dosage of pentamidine in these guidelines and the subsequent published reports on clinical trials and reviews. At present, only pentamidine isethionate is available, but the advised dosage for HAT and leishmaniasis is (historically) based on the amount of pentamidine base. In the treatment of leishmaniasis this is probably resulting in a subtherapeutic treatment. There is thus a need for a new, more transparent and concise guideline concerning the dosage of pentamidine, at least in the treatment of HAT and leishmaniasis.

## Antiprotozoal Activity of Pentamidine

**Figure 1:**
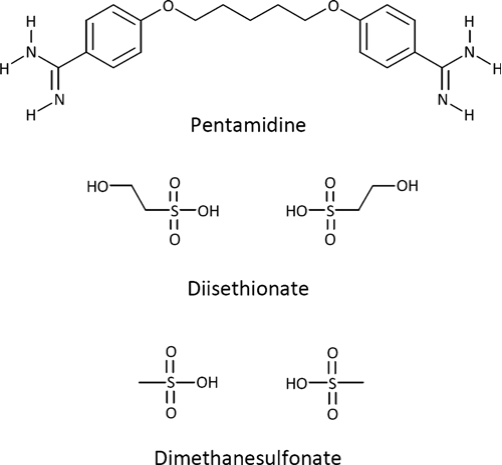
Structural Formulas of Pentamidine, Diisethionate, and Dimethanesulfonate.

The finding of the antiprotozoal activity of the diamidine family of drugs was largely a matter of serendipity. They were discovered during a search for hypoglycaemic compounds that could affect trypanosomes. Of the compounds synthesized, pentamidine (Figure 1) proved to be the most useful, and since the early 1940s it has been used in the treatment and prophylaxis of human African trypanosomiasis (HAT, also known as sleeping sickness) and to some extent in the treatment of visceral leishmaniasis in India. Nowadays, pentamidine is mainly used for prophylaxis and treatment of *Pneumocystis jirovecii* pneumonia (PCP), and in the treatment of first-stage HAT and of several forms of American cutaneous leishmaniasis.

In the past, two galenic formulations, both lyophilized salts of pentamidine (see Box 1), were available, one the 2-hydroxyethanesulfonic acid salt, called pentamidine isethionate (Pentacarinat or Pentam), the other the methanesulfonic acid salt, called pentamidine methanesulfonate or mesylate (Lomidine). These two preparations were used interchangeably, depending on local availability and preference, until the early 1990s when production of pentamidine methanesulfonate was stopped and only pentamidine isethionate remained.

**Box 1. Pentamidine Salts**

Because of the instability of aqueous pentamidine solutions, pentamidine (C_19_H_24_N_4_O_2_) is available for clinical use in the form of a powdered salt and reconstituted with water prior to administration. Pentamidine is a weak diprotic base due to the two amidine groups at both ends of the molecule, which means it can accept two protons in total and thus requires two monoprotic acid molecules to form a salt.

A few pentamidine salts are described in *The Merck Index* [18], which can be formed with the following acids:

- Hydrochloric acid, forming pentamidine dihydrochloride (C_19_H_24_N_4_O_2_.2HCl), which is not in clinical use at the moment.
- 2-Hydroxyethanesulfonic acid, forming pentamidine diisethionate (C_19_H_24_N_4_O_2_.2C_2_H_6_O_4_S, Figure 1), which is most often incorrectly described as “pentamidine isethionate”, also in *The Merck Index* [18].
- Methanesulfonic acid, forming pentamidine dimethanesulfonate or dimesylate (C_19_H_24_N_4_O_2_.2CH_4_O_3_S, Figure 1), which is also confusingly named “pentamidine methanesulfonate” or “pentamidine mesylate” in *The Merck Index* [18].

In accordance with most of the medical literature concerning pentamidine, the labels of the available preparations, and for reasons of uniformity, we chose to use the chemically incorrect names “pentamidine isethionate” and “pentamidine methanesulfonate” to refer to the two aforementioned salts of pentamidine in this article.

## Guidelines on the Treatment of Human African Trypanosomiasis

From the first published guidelines onwards, the dosage of pentamidine was expressed in and based on the active ingredient, the pentamidine base-moiety of the salt preparations. Pentamidine isethionate contains 1 g of base per 1.74 g of salt, while pentamidine methanesulfonate contains 1 g of base per 1.56 g of salt. The labelling of the ampoules of the different products is a source of confusion; pentamidine isethionate is labelled according to the amount of salt in the preparation (300 mg salt per ampoule), while pentamidine methanesulfonate was labelled according to the base-moiety (120 mg base per ampoule). The successive guidelines of the World Health Organization (WHO) for pentamidine in the treatment of *Trypanosoma brucei gambiense* infection as found in the WHO Technical Report Series demonstrate this confusion. In the first report of 1962, one reads that “3–4 mg of base/kg of body-weight” per injection should be administered [1].

In 1969 and 1979, the two different preparations are mentioned and it is indicated that the salts have to be back-calculated to the appropriate base-moiety such that 3–4 mg of base/kg body weight will be given [2],[3]. In 1986, confusion arose once more: again the two salts are mentioned, and it is indicated that 4 mg base/kg is to be administered, but the advised dose in millilitres per injection is based on the labelling of the different preparations and thus for pentamidine methanesulfonate on the base-moiety, but for pentamidine isethionate on the total amount of salt [4]. For pentamidine methanesulfonate this leads to the recommended dose of 4 mg base/kg per dose, but at the recommended 2 ml of pentamidine isethionate salt a 50-kg person only receives 200 mg of pentamidine isethionate salt per dose, which is equivalent to 2.3 mg base/kg, while at 4 mg base/kg 348 mg of isethionate salt should be given (1.74 mg isethionate salt = 1 mg base).

After interruption of production of pentamidine methanesulfonate, the confusion remained. In *Drugs Used in Parasitic Diseases*, a WHO monograph published in 1995, “4 mg/kg” per injection was advised without mentioning salt or base [5]. The WHO Expert Committee advised “4 mg of pentamidine isethionate per kg body weight” in 1998 [6], while the Scientific Working Group in 2003 recommended “4 mg of pentamidine base per kg” [7]. The latest clinical guidelines of Médecins Sans Frontières advise an ambiguous dosage of “4 mg/kg once daily” of pentamidine isethionate [8].

This confusion is also found in Manson's textbook of tropical medicine. In the 16th edition of 1966, both drugs are mentioned as salt and base and 4 mg base/kg is advised [9], but edition 17 of 1972 only mentions pentamidine isethionate and advises on 4 mg/kg per injection without mentioning salt or base [10]. This is continued in further editions. In recent publications on the treatment of trypanosomiasis, pentamidine [11] and pentamidine isethionate [12], both at 4 mg/kg per dose, are mentioned without indication of base or salt. As only pentamidine isethionate has been available since 1992, these authors probably treated patients with a relatively low dose of 2.3 mg base/kg per dose. Whether this low dose is a subtherapeutic dose and leads to a decrease in efficacy and possibly also in toxicity of pentamidine in HAT does not follow from these studies, but obviously needs further investigation. Indications of increased relapse rates since the change of pentamidine salts are not available. However, the lower dosage contradicts the latest WHO Scientific Working Group recommendation, and either this recommendation should be adjusted based on expert opinions and experiences from the field, or another dose should be applied [8].

## Pentamidine in the Treatment of Leishmaniasis

In the WHO Technical Report Series pertaining to the leishmaniases, 4 mg/kg is mentioned without indication of base or salt [13],[14]. An extensive review of the treatment of the leishmaniases does not mention the differences in composition and dosing of the two preparations [15]. Differences in efficacies are described for various types of leishmaniasis and for various regions, but without knowing the amount of actual product given, it remains difficult to assess the data [15]. After interruption of the production of pentamidine methanesulfonate, the differences between the two salts of pentamidine and their labelling and the resulting difference in efficacy rates were already noticed in the treatment of South American leishmaniasis [16]. In French Guyana, cutaneous leishmaniasis caused by *Leishmania guyanensis* was treated with 4 mg/kg pentamidine methanesulfonate (thus, 4 mg pentamidine base/kg). When, in 1992, pentamidine isethionate replaced pentamidine methanesulfonate and was used at the same dose of 4 mg/kg, clinicians noticed a decreased efficacy. A dose of 7 mg pentamidine isethionate/kg, equivalent to 4 mg pentamidine base/kg, restored effectiveness [16]. Also, in the treatment of visceral leishmaniasis in India, a difference in response to the two pentamidine formulations was noticed [17]. After the disappearance of pentamidine methanesulfonate from the Indian market, pentamidine isethionate was used at the “same” dosage of 4 mg/kg, and a reduction in efficacy as well as toxicity was observed [17]. Most probably, the dosage was based on the labelling and thus only 2.3 mg/kg base (4 mg/kg pentamidine isethionate) was administered. Retrospectively, the observed higher relapse rate and less distinct toxicity profile in these visceral leishmaniasis patients was not caused by a different kind of pentamidine salt, but by an inherent, unnoticed change of dosage.

## Conclusion

There is a need for a more transparent guideline concerning pentamidine, at least for the treatment of HAT and leishmaniasis. The former availability of different salts of pentamidine and their differences in the amount of active ingredient and labelling of the preparation led to great confusion in guidelines and reviews, and reports on clinical trials. In the future, it should clearly be stated which preparation is used and on which moiety of the preparation (either salt or base) the mentioned dosage is based. Since only pentamidine isethionate is available at the moment and this preparation is labelled according to the amount of salt, a scheme involving the volume of pentamidine isethionate solution to administer per kilogram of body weight seems most rational as a practical guideline for nurses and clinical officers. This should rule out any inconsistent dosing determining falsely the dose–efficacy relationship and will make future clinical trials better comparable.
